# Unfulfilled need for contraception among women with unmet need but with the intention to use contraception in Rakai, Uganda: a longitudinal study

**DOI:** 10.1186/s12905-018-0551-y

**Published:** 2018-04-27

**Authors:** Tom Lutalo, Ron Gray, John Santelli, David Guwatudde, Heena Brahmbhatt, Sanyukta Mathur, David Serwadda, Fred Nalugoda, Fredrick Makumbi

**Affiliations:** 1 Rakai Health Sciences Program, Uganda Virus Research Institute, P.O Box 49, Entebbe, Uganda; 2 Department of Epidemiology, Johns Hopkins Bloomberg School of Public Health, Baltimore, MD USA; 3 Joseph L Mailman School of Public Health, Columbia University, New York, USA; 4 Makerere University School of Public Health, Kampala, Uganda; 5 Department of Population, Family and Reproductive Health, Johns Hopkins Bloomberg School of Public Health, Baltimore, MD USA

## Abstract

**Background:**

Longitudinal data from a rural Ugandan cohort was used to estimate rates of unfulfilled need for contraception, defined as having unmet need and intent to use contraception at baseline but having an unintended pregnancy or with persistent unmet need for contraception at follow up.

**Methods:**

Between 2002 and 2009 (5 survey rounds), a total of 2610 sexually active non-pregnant women with unmet need for contraception at the start of an inter-survey period were asked whether they intended to use any method of contraception until they desired a child. Modified Poisson multivariate regression was used to estimate unadjusted and adjusted prevalence ratios (PR) and 95% CI of unfulfilled need for contraception.

**Results:**

The proportion of women with unmet need at the start of an interval who intended to use contraception significantly increased from 61 to 69.1% (*p* < 0.05). However the majority of women who said they intended to use contraception had unfulfilled need for contraception at the subsequent survey (64.8 to 56.8%). In the adjusted analysis, significant predictors of unfulfilled need for contraception included age 40–49 years (PR = 1.34; 95% CI 1.04–1.74) and those with unknown HIV status (PR = 1.16; 95% CI 1.06–1.26).

**Conclusions:**

There is a significant discrepancy between women’s intent to use contraception (> 60%) and subsequent initiation of use (< 30%) with many having unintended pregnancies which might explain the persistent high fertility in Uganda. Future research needs to address unfulfilled need for contraception among women at risk of unintended pregnancies.

## Background

Unmet need for Family Planning (FP) has served as an organizing concept for population policies for over 25 years [1]. Addressing unmet need is consistent with human rights and a feminist approach to fertility control, and it has been estimated that the fertility reduction goals of most programs could be achieved by meeting the contraceptive needs of women who desire to avoid or postpone future births [2]. Reductions in unmet need would reduce the occurrence of unwanted pregnancies, meet the fertility desires of many women and serve national population policy goals. There have been major reductions in unmet need in most regions of the world with the exception of sub-Saharan Africa where levels of unmet need, at ~ 26%, remain unacceptably high among married fecund women who do not want to become pregnant [3]. While unmet need for FP serves as an organizing concept for population policies it does not necessarily reflect latent demand for contraception [4]. The relationship between ‘unmet need’ and “unfulfilled need to use contraception” needs to be studied in settings where lack of resources and other barriers affect women’s reproductive choices. The term “unfulfilled need” implies that women with an unmet need may intend to use contraception in the future, but may fail to initiate contraception due to limited choices if services are unavailable. Unwanted or unplanned pregnancies among women with unmet need who intend to use contraception, leads to the notion of unfulfilled need for FP. A study among women within one year of their last birth found that two thirds had unmet need for contraception and nearly 40% said they planned to use a method in the next 12 months, but were currently not doing so [5]. An earlier study in 25 developing countries found that about half the women with unmet need had no intention of using contraceptives even if they were made freely available [6].

This discrepancy between unmet need, stated intention to use contraception and failure to initiate contraception poses a challenge to the prevention of unintended pregnancies, reduction of unplanned births and unsafe abortions, improvement of maternal and child health and slow population growth [7–9].

Most studies of unmet need and subsequent use of contraception were cross-sectional and restricted to married women not using family planning. We assessed the relationship between unmet need and unfulfilled need for contraception among women who state that they intend to use family planning, using a longitudinal approach.

Uganda has one of the highest annual population growth rates in the world, at 3% per annum, and the highest Total Fertility Rate (TFR) of 5.4 children per woman in East Africa [10]. The Uganda Demographic and Health Survey of 2016 showed that only 35% of currently married women report use of modern contraception and 28% have an unmet need for FP.

The objective of this analysis was to estimate rates and factors associated with unfulfilled need for contraception in a population cohort of sexually active women of reproductive age with an unmet need for spacing or limiting child bearing, who expressed the intention to use contraception.

## Methods

The data came from the Rakai Community Cohort Study (RCCS), a prospective, open-cohort of consenting adolescents and adults aged 15–49 years interviewed at approximately 12–16 month intervals, in 50 communities in Rakai District, South Central Uganda. The RCCS has been described elsewhere [11, 12]. The RCCS survey includes all consenting residents (men and women), aged 15–49 years (n ~ 14,000 per survey round) and each RCCS participant receives a unique, life-long identification number used to link data over time. Participants sign a written informed consent for study participation, sample collection and HIV testing. Experienced same-sex interviewers, fluent in the local language, have been trained to ensure participant privacy and confidentiality. Using data from five survey rounds between 2002 and 2009 (survey rounds 9 to 13; 2002/03, 2003/04, 2005/06, 2006/08, 2008/2009 respectively), participants were administered a structured questionnaire to assess socio-demographic characteristics, sexual risk behaviors, fertility desires and intentions, pregnancy history and contraceptive use. During survey rounds 9 to 12 a question on intention to use any method of contraception in the future was asked of female participants. The question was “Do you intend to use any contraceptive method between now and your next pregnancy?” Responses included;-“Yes, No, Family Planning method already mentioned, Do not Know”. The family planning methods included both modern (oral contraceptives, male condoms, spermicides, injection, Intrauterine Device, tubal ligation and implants) and traditional methods (abstinence, calendar/rhythm, breast feeding, herbs/traditional medicines and other specified methods which included withdrawal). During survey round 13 (2008/2009) we identified women with unmet need for FP.

### Definitions of variables used

#### Unmet need for contraception

Was defined as women who did not want to have a child in the next two years (spacers) or to have no more children (limiters) and were not using any method of contraception [13]. All sexually active women who had unmet need for spacing or limiting childbirth were included in the study. Women who were not pregnant at the time of the survey, were asked whether they planned to use contraception until they wanted to have a child.

#### Unfulfilled need for contraception

Was defined as non-pregnant women with unmet need for contraception who planned to use a method of contraception in the future but at follow-up either reported an unwanted pregnancy during the inter-survey period or still had an unmet need for contraception at the subsequent survey. Women who had an unwanted pregnancy included those with a completed pregnancy between surveys and those who were found to be pregnant at the follow up survey who reported they did not want to have that pregnancy. Women with a completed pregnancy between surveys were asked “Did you intend to have that pregnancy at that time?” while women who were found with a pregnancy were asked “Did you intend to have this pregnancy at this time?” Those women whose answer was a “No” to the above questions were asked “Was that/this pregnancy just mistimed, totally unwanted or you did not care whether it happened or not?” It is those women whose response was “totally unwanted” that were categorized as having an unwanted pregnancy. Women who reported that the pregnancy was mistimed or that they did not care were not categorized as having an unwanted pregnancy.

#### Survey visits considered for this study

Only women who had at least two survey visits were considered for this study. The first time the woman was included was considered a baseline survey and the subsequent survey was considered a follow up. Some women had one follow up survey while others had two or more follow up surveys after the baseline.

The analysis excluded pregnant women at the start of a follow-up period.

### Statistical analysis

Modified Poisson multivariable regression with robust variance was used to estimate unadjusted and adjusted prevalence ratios (PR) and 95% confidence intervals (95% CI) of unfulfilled need for contraception, accounting for clustering due to repeated observations across surveys. Factors found to be associated with unfulfilled need with a *p*-value of < 0.2 in bivariate analyses as well as potential confounders (place of residence, age, marital status, education, number of living children, household wealth index and HIV status) were included in multivariate models [14, 15].

## Results

A total of 2610 sexually active women with unmet need for contraception provided data for this analysis. Of these women 1741 (66.7%) had one follow up, 623 (23.9%) had two follow up surveys, 188 (7.2%) had three follow up surveys and 58 (2.2%) had four follow up surveys providing a total of 6393 person observations. The main reason for the loss to follow up was participant absence or out migration from the study community.

Women were categorized as having unmet need for contraception who either did or did not express an intention to use contraception. The majority of women lived in rural communities (> 80%), were married, had more than 3 living children and resided in households with a low wealth index (Table 1). 35–42% were between 20 and 29 years of age, but there were significant increases in age over time. The proportion of HIV-negative women also increased over time.

**Table 1:** Socio-demographic and behavioral characteristics of the women at the beginning of an inter-survey period (2002–08)

	Survey Periods				
Characteristic	Round 9	Round 10	Round 11	Round 12	*p*-value
	2002/03	2003/04	2005/06	2006/08	
N	1030	981	978	794	
Residence	%	%	%	%	
Rural	87.2	83.0	85.2	87.2	0.026
Urban	12.8	17.0	14.8	12.9	
Age Years					
15–19	4.6	2.8	1.7	1.1	< 0.001
20–29	41.8	41.0	40.3	35.3	
30–39	29.5	30.8	32.8	38.5	
40–49	24.2	25.5	25.2	25.1	
Marital Status					
Currently married	81.4	79.8	79.0	82.4	0.312
Never Married	4.7	6.2	6.2	4.2	
Div/Sep/Widowed	14.0	14.0	14.7	13.5	
Education					
No education	11.2	12.3	13.0	11.9	0.469
Some primary	73.2	69.5	68.9	70.3	
Post Primary	15.6	18.1	18.1	17.8	
Number of living children					
None	1.1	0.71	1.1	0.7	0.021
1–2	23.9	21.1	22.0	16.0	
3–5	41.6	43.4	43.7	46.1	
6+	33.5	34.8	33.2	37.3	
Household Wealth Index					
Low	45.2	43.9	43.4	42.7	0.844
Medium	28.5	27.5	29.2	30.0	
High	26.3	28.5	27.4	27.3	
HIV Status					
Positive	8.3	10.2	10.8	8.9	< 0.001
Negative	67.4	73.2	81.5	89.0	
Unknown	24.4	16.6	7.8	2.0	

### Proportion with unmet need for contraception but with an intention to use contraception

Overall the proportion of women with unmet need at the start of the interval who intended to use contraception significantly increased from 61% in 2002/03 to 69.1% in 2006/08 (*p* < 0.05; Table 2). This increase was only observed among married women but not among the never married and previously married women.

**Table 2:** Intention to use contraception by marital status and survey round among women with unmet need for contraception

Marital Status	2002/03	*Survey* 2003/04	*Period* 2005/06	2006/08	*p*-value
*Overall*					
With unmet need (N)	1,030	981	978	794	<0.05
Intends to use contraception (%)	61.0	63.5	63.8	69.1	
Does not intend to use contraception (%)	39.0	36.5	36.2	30.9	
*Married* With unmet need (N) Intends to use contraception (%) Does not intend to use contraception (%)	838 63.3 36.8	783 64.6 35.4	773 65.7 34.3	654 71.4 28.6	<0.05
*Never Married* With unmet need (N) Intends to use contraception (%) Does not intend to use contraception (%)	48 70.8 29.2	61 77.1 23.0	61 73.8 26.2	33 78.8 21.2	0.831
*Previously married* N Intends to use contraception(%) Does not intend to use contraception (%)	144 44.4 55.6	137 51.1 48.9	144 49.3 50.7	107 52.3 47.7	0.590

The majority of women who intended to use contraception at the start of a follow up period were found to have unfulfilled need for contraception by the subsequent survey (56.8–64.8%; Table 3). This suggests the majority of women with unmet need who intended to use contraception did not use contraception or had an unwanted pregnancy or persistent unmet need (i.e., unfulfilled need for contraception). However, the proportion of those with unfulfilled need for contraception declined significantly from 64.8% in 2002/03 to 56.8% in 2008/09 (*p* < 0.01; Table 3). This decline was only observed among the married women (data not shown).

**Table 3:** Intention to use contraception^b^ by reported use at the subsequent survey round

Intention to use contraception at start of interval	Status by/at Round 10 (2003/04; row %)*				
	N	No need for Contraception^a^	Uses FP	With Unmet Need	Unfulfilled Need
Round 9 (2002/03)					
Intends to Use	628	11.7	23.6		64.8
Does not intend to use	402	10.7	7.2	82.1	
Round 10 (2003/04)	Status by/at Round 11 (2005/06; row %)*				
	N	No need for Contraception	Uses FP	With Unmet Need	Unfulfilled Need
Intends to Use	623	15.8	25.0		59.2
Does not intend to use	358	12.6	5.9	81.6	
Round 11 (2005/06)	Status by/at Round 12 (2006/08; row %)*				
	N	No need for Contraception	Uses FP	With Unmet Need	Unfulfilled Need
Intends to Use	624	14.4	26.9		58.7
Does not intend to use	354	12.4	9.9	77.7	
Round 12 (2006/08)	Status by/at Round 13 (2008/09; row %)*				
	N	No need for Contraception	Uses FP	With Unmet Need	Unfulfilled Need
Intends to Use	549	14.9	28.3		56.8
Does not intend to use	245	10.6	11.8	77.6	

*p < 0.001

^a^Had/has wanted pregnancy or Desires for a child in the next two years

^b^Intends to use any method of contraception until when she desires for a child

Women with unmet need at the start of an interval, who had no intention of using contraception had a high proportion of unmet need for contraception by the subsequent survey compared to those who intended to use contraception (Table 3). This ranged from 82.1% in 2003/04 to 77.6% in 2008/09.

### Factors associated with unfulfilled need for contraception

The unadjusted and adjusted prevalence ratios of factors associated with unfulfilled need for contraception are shown in Table 4. In the unadjusted analysis, significant predictors of unfulfilled need included age (> = 30 years) and women of unknown HIV status. Women with higher education and households with a high wealth index were less likely to have unfulfilled need for contraception. In the adjusted analysis, significant predictors of unfulfilled need included age group 40–49 years (PR = 1.34; 95% CI 1.04–1.74) and those with unknown HIV status (PR = 1.16; 95% CI 1.06–1.26).

**Table 4:** Unadjusted and adjusted Prevalence Ratios of unfulfilled need for contraception

Characteristic	Unadjusted		Adjusted	
	PR	95% CI	PR	95% CI
Residence				
Rural	1		1	
Peri-urban	0.93	0.84–1.03	1.00	0.91–1.11
Age Group				
15–19	1		1	
20–29	1.13	0.90–1.41	1.11	0.87–1.40
30–39	1.32	1.05–1.65	1.22	0.96–1.57
40–49	1.50	1.19–1.89	1.34	1.04–1.74
Marital Status				
Never Married	1		1	
Married	0.88	0.76–1.03	1.04	0.89–1.22
Ever Married	0.99	0.88–1.10	1.02	0.85–1.22
Number of living children				
None	1		1	
1–2	1.77	0.68–4.59	1.82	0.73–4.54
3–5	1.90	0.74–4.91	1.83	0.80–4.58
6+	2.31	0.89–5.95	2.02	0.90–5.08
Education				
None	1		1	
Some Primary	0.88	0.80–0.97	0.94	0.85–1.04
Post Primary	0.80	0.71–0.90	0.90	0.78–1.02
Household Wealth Index				
Low	1		1	
Medium	0.94	0.87–1.02	0.97	0.90–1.05
High	0.89	0.82–0.97	0.94	0.86–1.03
HIV Status				
Negative	1		1	
Positive	1.01	0.91–1.13	1.02	0.91–1.14
Unknown	1.16	1.07–1.27	1.16	1.06–1.26

## Discussions

Our longitudinal study assessed unfulfilled need for contraception among women in rural Uganda and found a discrepancy between women’s stated intent to use contraception (> 60%) and the proportion of women who initiated contraceptive use (< 30%) at follow up. Intention to use contraception was associated with subsequent use of contraceptives among married (> 60%), and never married women (> 73% at each survey round).

Persistent unmet need among women who had initial unmet need with no intentions to use contraception was > 77% at each subsequent survey. There is a need to develop strategies targeting women with unfulfilled need for contraception and promote contraception among those with no intention to use contraception. Demographic and Health Surveys in 52 developing countries between 2005 and 2014 found that 26% cited concerns about contraceptive side effects and health risks; 24% say they had sex infrequently or not at all and 23% say that they or others close to them oppose contraception [9].

The results also suggest high rates of unfilled need for contraception among women aged 40–49 years and those with unknown HIV status in this population. Those with unknown HIV status may not have received HIV counseling which is significantly associated with use of contraception and decreases in unmet need [16].

There are limitations to this study. The data were not specifically collected for the primary study objectives and the results may not be representative of Uganda.

The data were collected some time back meaning there is a need for a similar study now for comparison purposes. The study did not control for some potentially predictive factors like mobility (e.g. outmigration). Self-reported intentions are also subject to recall or social desirability bias and misreporting. Additionally, the study did not capture changes in intention to use or actual use of contraception during inter-survey periods but only considered reported use at the time of the interview. We also did not know the partner’s influence on contraceptive decision making or the availability/accessibility of contraceptive services. However over 98% of these women knew where to access contraceptives.

## Conclusions

The study, which targeted women with unmet need who expressed an intention to use contraception showed that many did not subsequently adopt contraception. This group of women constitute a high priority group and have great potential to increase the cost effectiveness of future expansion of family planning programs. Further studies are needed to assess what are the reasons for their failure to adopt family planning.

## Abbreviations

CI: Confidence Interval
DHS: Demographic and Health Survey
FP: Family Planning
HIV: Human Immunodeficiency Virus
PR: Prevalence Ratio
RCCS: Rakai Community Cohort Study
TFR: Total Fertility Rate
